# Health Professionals Facing Suicidal Patients: What Are Their Clinical Practices?

**DOI:** 10.3390/ijerph15061210

**Published:** 2018-06-08

**Authors:** Inês Rothes, Margarida Henriques

**Affiliations:** Center for Psychology, Faculty of Psychology and Education Sciences, University of Porto, Porto 4200-135, Portugal; mrangel@fpce.up.pt

**Keywords:** health professionals, suicidal patients, clinical practices

## Abstract

Clinical work with suicidal people is a demanding area. Little is known about health professionals’ practices when faced with suicidal patients. The aims of this study were to: (1) describe the practices most likely to be adopted by professionals facing a suicidal patient and (2) analyze the differences according to professional characteristics (group, specific training on suicide, and experience with suicidal patients). A self-report questionnaire that was developed for this study was filled out by 239 participants. Participants were psychologists, psychiatrists, and general practitioners who work in different contexts: hospitals, public health centres, schools or colleges, and community centres. Principal components analysis, analyses of variance, and *t*-tests were used. Four components were identified: (1) Comprehensive risk assessment; (2) protocols, psychotherapy and connectedness; (3) multidisciplinary clinical approach; and, (4) family, explaining a total of variance of 44%. Positive associations between suicide-related variables (training and experience) and practices were found. In general, health professionals’ practices are evidence-based, however a relevant percentage of professionals can benefit from training and improve their practices.

## 1. Introduction

According to the World Health Organization, about one million people die by suicide every year worldwide, which makes suicide prevention one of the most important challenges of public and mental health [1,2]. Research showed high rates of contact of suicidal people with health professionals before the suicidal act, both in fatal [3] and non-fatal behavior [4]. This contact of an individual at suicide risk with health services is an opportunity to prevent it. Health professionals can be an important help to prevent suicidal behaviors, but at same time, research has been demonstrating the existence of different barriers that can hinder the pivotal role that health professionals can have in risk detection, assessment, and intervention with suicidal patients [5,6,7]. When facing a patient who has suicidal problems, health professionals have to perform a competent clinical assessment of suicide risk [8,9] and they have to make choices and decisions regarding the care to provide, which can have significant consequences to the person at risk [10]. Health professionals reported difficulties in the clinical work with suicidal patients including a lack of knowledge about suicidality and about effective interventions [11]. Difficulties that were reported by the health professionals included different types of issues: technical, emotional, relational, communicational, and related to the family approach [11].

These concerns have generated a growing body of research focused on health professionals’ characteristics, attitudes, and behaviors, as variables potentially related to these barriers [7,12,13,14]. In this regard, previous studies investigated the attitudes of health professionals towards suicidal patients [13]; the knowledge and skills on suicidal management, including the self-perception and confidence in competences [7,12]; clinician’s characteristics and other factors that influence the assessment of risk, as, for example, the legal liability [5,15]; the difficulties of health professionals [11]; the explanations of health professionals for suicidal behaviors [16,17]; and, the comfort and fear in working with suicidal patients [14]. But, little research was conducted on the practices of health professionals towards suicidal behaviors. To our knowledge, only few studies about health professionals’ suicide risk assessment and management practices are available. Namely, there are: (1) some studies about suicide risk assessment procedures [8,18]; (2) a set of studies addressing the use of no-suicide contracts [19,20,21]; and, (3) one very recent study about the frequency with which mental health professionals use different suicide risk assessment and management practices [14]. (1) Findings from the studies about risk assessment procedures indicated the existence of failures in recognizing suicide risk among health professionals [18,22,23,24] and identified that clinicians preferred the use of interview questions rather than suicide assessment instruments [8]. (2) Studies about the use of no-suicide contracts (NSC) indicated that a high percentage of health professionals use NSCs, having no specific training and thus enhancing the likelihood of potential damages of the use of this tool [20,21,25]. (3) Finally, a recent study from Roush et al. [14] identified that over 30% of mental health professionals did not ask every patient about suicidal thoughts or behaviors in first visits, but when facing suicidal patients, the majority of mental health professionals (between 68 and 77%) reported conducting a suicide risk assessment. Nevertheless, this study did not address how suicide risk was assessed. The use of other adequate practices was explored—asking about lethal means was reported by 34% of the clinicians and the inclusion of the family in the process was reported by 40% [14].

These studies addressed important questions about practices of health professionals towards suicidal patients. However, the use of other clinical approaches with strong or promising evidence of effectiveness in reducing suicidality remains insufficiently studied. Concretely, what are the actual clinical practices and questions regarding risk assessment, the actions targeting the patient's family connection, the options regarding the treatment of co-occurring mental health problems and other difficulties, as well as the option that is related to the chain of care or the multidisciplinary approach, remain basically unknown. 

Different clinical interventions in suicide risk show strong evidence of efficacy for reducing death by suicide and/or the repetition of suicidal behaviors. These interventions include: fostering connectedness using phone and technology-based methods (e-mail and texting) [26,27]; reducing access to lethal means [28]; and, treatment of co-occurring mental disease, including pharmacotherapy [29] and psychotherapy, namely cognitive behavioral therapy [30].

Given the scarcity of research in this specific topic of the practices of health professionals towards suicidal patients and the valuable role that clinicians can play in suicide prevention, this study investigates the clinical practices of psychologists, psychiatrists, and general practitioners (GP) when a suicidal patient seeks their help. Understanding professional practices is a fundamental step toward improving training and competence in this field [31]. The health professionals most sought by people who have psychological or emotional problems are psychiatrists, GPs, and psychologists [32]. In Portugal, study setting, the access to health care is universally free of cost (free or low fees). In recent years, however, the private health system has developed and increased, especially in large urban centres. Most of the population have a GP of reference in the public health system—in public centres of primary health care. The public psychiatric care is mainly integrated into the mental health and psychiatric services of hospitals. But, a considerable part of the population that uses psychiatric care uses the private system, private clinics, and hospitals. Psychologists work both in health centres and in public hospitals, but they are still insufficient at the level of the public health service. There is also free psychological care at community centres/services. Private practice is also common for psychological care.

The goals of this study were (1) to describe the current practices of psychologists, psychiatrists, and general practitioners towards suicidal patients, (2) to identify the differences in practices according to the professional group, and (3) to test the existence of differences in practices due to health professionals’ characteristics, namely having or not specific training in suicide intervention and having different levels of experience with suicidal patients. Previous studies have found differences in attitudes, skills, and difficulties, according to specific suicide-related variables, such as experience with suicidal patients and specific training on suicide intervention [7,11,12]. Consistent with these previous studies, it was expected that training and specific clinical experience with suicidal behaviors would positively influence professionals’ practices. Thus, the following hypotheses were formulated: (1) Health professionals with specific training in suicide have a higher tendency to adopt adequate practices than health professionals without specific training; and, (2) health professionals with more experience in suicidal behaviors have a higher tendency to adopt adequate practices than those with less experience. 

## 2. Materials and Methods 

### 2.1. Survey Instrument Development—Intervention Strategies towards Suicidal Behaviors Questionnaire—ISBQ—Version for Psychologists and Doctors

To identify the current practices of health professionals towards a suicidal patient, a questionnaire was developed. The ISBQ was based on the results of a previous qualitative study conducted with 30 professionals (psychologists, psychiatrists, and general practitioners) [33]. In this previous study, the free association technique was used as a method for data collection, in order to gather the spontaneous responses of participants regarding practices towards a patient who attempted suicide. To select the ISBQ items based on the answers from health professionals, frequencies analysis and correspondence factor analysis were used. ISBQ items were based on the most frequent and outgoing answers of health professionals and on literature in the area. The ISBQ resulted in 40 items each scored on a five-point Likert scale from 1 (not likely at all) to 5 (very likely of being adopted as practice with a patient that seeks help following a recent suicide attempt). The heading was the same for all the items: “*Choose to what extent it is likely or not that you adopt the following intervention strategies with a patient that seeks your practice following a recent suicide attempt. Even though clinical practice varies depending to each case we would like you to answer according to your general practice*”. The questionnaire also collects information about training on suicide prevention, suicidal patient experience, and socio-demographic data. The ISBQ is available below as Table A1. ISBQ showed good psychometric characteristics, albeit preliminary (cf. results section). 

### 2.2. Participants and Procedures

Prospective participants—psychologists, psychiatrists, and general practitioners—were approached in order to gather participants from the entire country (Portugal) and from different workplaces. A combination of sampling methods was used: targeted sampling [34] and snowball sampling [35,36]. In the first technique, target work settings were identified (such as hospitals, health centers, and community intervention centers), according to a previous geographical mapping. In each of these settings, a presentation of the research was carried out (in presence, by email or letter) in order to invite the professionals to participate. Associations of psychotherapy and the Portuguese Society of Suicidology were also contacted and their members were invited to participate in the survey. The second sampling method, snowball, is characterized by participants recruiting other potential participants through their professional or personal networks. An additional set of methodological procedures, recommended in literature, was used. These procedures are presented as having advantages compared to the traditional snowball in the selection of the sample: (1) professionals helped recruiting in two ways: they directly invited other colleagues to participate and they designated prospective participants, sending their contacts to the researchers; (2) part of the participants had the number of professionals that they could recruit limited to 3–5 colleagues; and, (3) a set of inducements to promote participation was used (customized letter, email or phone call appealing for participation and mentioning the colleague who had indicated him/her) [37]. The additional set of procedures brings an advantage in relation to traditional snowball, promoting the composition of samples that converge and reach equilibrium after a relatively limited number of recruitment chains, independently of the initial sample (the seeds). This way, despite the non-random selection, the bias that is introduced is progressively eliminated. Moreover, the additional procedures reduce the biases resulting from differences in the size of personal networks and from the designated voluntarism [38].

Data were collected through both a web survey and a mail survey. Anonymity and confidentiality of data collected were guaranteed. The study protocol was approved by the Portuguese Society of Suicidology and by the Ethics Committee of the Hospital Centro Hospitalar do Nordeste, E.P.E., Bragança, Portugal. 

### 2.3. Data Analysis

Statistical analyses were carried out using SPSS version 21 (IBM, Armonk, NY, USA). Descriptive statistics were calculated. Principal components analysis (PCA) was performed in order to reduce the large number of items to a more manageable number. Component loadings of at least 0.38 were considered meaningful based on guidelines for identifying significant factor loadings based on the sample size [39]. To determine the number of components, a parallel analysis was conducted and different possible solutions were explored. Oblimin was used to rotate the components to a simple structure and to obtain oblique solutions, allowing for factor correlations. The reliability (internal consistency) was calculated by Cronbach’s alpha and by the mean inter-item correlation. Component scores were obtained by the mean of items with a meaningful loading on the respective components.

Differences in practices between professional groups and according to professional characteristics (having specific training on suicide and experience with suicidal patients) were determined by one-way between-groups multivariate analysis of variance (MANOVA). Preliminary assumption testing was conducted with no serious violations noted. Additionally, to verify if professional group moderated the relationship between specific training in suicide prevention and the practices, two-way between groups ANOVA tests were carried out.

The experience with suicidal patients was measured through the number of patient suicide attempts that were reported by professionals and re-coded into interval categories: many (≥9), moderate (4–8), and few (≤3) patients suicide attempts.

The relationship between professional group and specific training, and the relationships between professional group and referring/advising to specialized counseling/monitoring (psychiatric, psychological, by GP) were investigated by chi-squared tests. 

Missing data ranged between 2 to 5%. Given the low values of missing data, the use of traditional technique of pairwise deletion was considered to be suitable to prevent sample size reduction [40].

## 3. Results

### 3.1. Participants Characteristics 

A total number of 242 health care professionals filled out the questionnaire, but three were eliminated because of a high level of missing answers, resulting in 239 participants. Participants’ characteristics are reported in Table 1. The age of these professionals ranged from 23 to 77 years (M = 38.5, SD = 11.8). With regard to years of experience, the sample ranged from health professionals who were starting their careers to professionals with many years of experience (range 0–48 years M = 13.1, SD = 10.8) and the majority of the health professionals (79.5%) had experience with suicidal behaviors in clinical practice. 

Work places are varied, about 11% worked in more than one location and 44% accumulated these places with private practice.

Of the health professionals who reported having specific training, 79.1% reported having training in suicide risk assessment, the same percentage in crisis intervention, 41.9% in epidemiology, and 34.9% in forensic sciences. Eighty-five percent (85%) of the health professionals rated themselves as able to assess suicide risk and 43% rated their training as sufficient to deal with suicidal patients in their practice.

### 3.2. Practices of Health Professionals 

The questions most commonly rated by health professionals as probable or very probable to be asked to suicidal patients were about: current problems (92%), the existence of a suicide plan (84%) and prior attempts (84%), alcohol and drugs use (77%), family suicide background (74%), the desire to die (73%), and about the lethal means used by the patient (70%). Among the practices, the most likely to be used by a large proportion of health professionals, were also the exploration of the meanings and triggers of the act (93%) and the circumstances of the attempt (83%). Trying to understand the style of coping of the patient was also considered as likely or very likely to be adopted by a significant proportion of participants (72%), as well as the exploration of expectations (71%) and the feelings about having survived (62%). Depression assessment was indicated by 93% of the health professionals as a probable or very probable procedure, while the evaluation of hopelessness was considered with high likelihood to be carried out by 66% of the participants. Referring to a psychiatrist was rated by 84% of the participants as probable or very probable, while a referral to a psychologist by 76%. With regard to referring/advising to psychiatric counselling, significant differences were found between professional groups (χ^2^ = 26.34, df = 4, *p* = 0.000). General practitioners were the professional group who most refers/advises to psychiatry (98%), followed by psychiatrists (87%). Psychologists were the group who least advises psychiatry (75%). There were also significant differences between professional groups regarding psychological counselling (χ^2^ = 23.80, df = 4, *p* = 0.000). Psychiatrists were the group who least refers to psychological counselling (58%) and psychologists were who most advises (83%). The proportion of GPs who rated to advise to psychological counselling as probable or very probable was 75%.

A continued care plan, regardless of the caregiver’s specialty, was rated by 90% and psychotherapy was considered by 74% of the health professionals as probable or very probable.

Regarding family involvement, engaging family in the process was considered to be probable or very probable by 60%, to provide family support by 43% and to conduct an interview to the family by 41%. In contrast, among the practices or strategies that are classified as less probable to be used by health professionals, were using the internet to communicate (which 7% rated as likely or very likely to use) and using written no-suicide contracts, which 22% consider to be likely or very likely, 15% as somewhat likely, and 62% rated as unlikely to be used. Among the less probable practices to be adopted were also the use of intervention and assessment instruments (three items: 23, 26, and 31%) and giving the mobile phone number (33%). Referring to a GP was rated by 20% of the health professionals as probable or very probable, and inpatient care by 26%. There were also significant differences between professional groups regarding to refer/advise monitoring by GP (χ^2^ = 32.27, df = 4, *p* = 0.000). The proportions of psychiatrists and psychologists who refer to GP were 5.7% and 14%, respectively, while the proportion of GPs who advises this monitoring was 45%. Table 2 shows the frequencies of practices likely and very likely to be adopted by psychologists, psychiatrists, and GPs towards suicidal patients.

To determine the final number of factors, the retention of four, five, or six factors was explored. The most theoretically interpretable and consistent solution consisted of 39 items into four factors, explaining a total variance of 44%. In Table 2, the Oblimin-rotated results are shown. The Cronbach alpha coefficients and the mean inter-item correlation revealed a satisfactory internal consistency. 

The items with meaningful loadings on more than one component were assigned to the one on which they loaded highest. One item was eliminated because its removal increased reliability of the factor, changing the reliability from 0.913 to 0.915. Although the item “*I suggest using the internet to communicate*” had a lower factor loading than the meaningful value considered (0.38), it was kept because of the importance of its content, namely relating to the effectiveness evidence of regular contacts for suicidal behaviors prevention [26].

The first component was named “Comprehensive risk assessment” and contains 21 items corresponding to a wide set of actions, including the evaluation of a crisis situation or imminent risk, intentionality, lethality, triggers, and risk factors (e.g., previous suicide attempt, drugs and alcohol use, eventual mourning, and other current problems). It also includes coping style, depression, and hopelessness assessment. The mean of the percentages of the practices rated as likely or very likely to be adopted of the 21 risk assessment items was 75% (range 38–93%) and the mean score of the component was M = 4.12 SD = 0.57 (range 4.66–3.13). 

The second component was labelled “Protocols, psychotherapy and connectedness” and includes eight items corresponding to the use of formal instruments in the evaluation process and intervention, including the use of written contracts. This component comprises the referral to psychotherapy and also strategies fostering connectedness, using the phone and internet. The mean of the percentage of the practices rated as likely or very likely to be adopted for the eight items of this component was 34% (range 7–74%) and the mean score of the component was M = 2.82, SD = 0.76 (range 1.73–4.10).

The third component called “Multidisciplinary clinical approach” comprises seven items, including following by psychiatrist, psychologist, and general practitioner, highlighting the specialized and continuous monitoring, and also containing pharmacotherapy and inpatient care treatments. The mean percentage of the practices rated as likely or very likely to be adopted for the seven items of this component was 59% (range 20–93%) and the mean score of the component was M = 3.68, SD = 0.58 (range 2.28–4.51).

The fourth component called “Family” comprises three items about the involvement of the family in the process of assessment and management of suicide risk and the support that is provided to the family of the suicidal patient. The mean percentage of the practices rated as likely or very likely to be adopted for the three items of this component was 48% (range 40%–60%) and the mean score was M = 3.45, SD = 0.87 (range 3.25–3.78). 

With regard to psychometric characteristics, ISBQ presented a good sensibility-answers to 32 items ranged from the lowest to the highest value (1 to 5), answers to five items that were scored from 2 to 5, and answers to two items scored from 3 to 5; a favorable exhaustiveness—the items inductively generated, suggest the exhaustiveness; a good factorial validity—the first—order principal components analysis identified four factors and the items have a meaningful loading on the components where they fit well; a satisfactory internal consistency—in three of the four factors the Cronbach’s alpha coefficient ranges from 0.92 to 0.79, and in the other one (with alpha below 0.7), the mean inter-item correlation is 0.2, i.e., between the optimal value recommended (0.2 to 0.4); and, a favorable construct validity—discriminates assessment practices between professionals with and without specific training on suicide and between professionals with different rates of suicide attempters in clinical practice.

### 3.3. Factors Related to Practices

#### 3.3.1. Professional Group

There was a statistically significant difference between professional groups on the combined dependent variables (intervention practices with suicidal people): F (8, 468) = 25.61, *p* = 0.000; Pillai’s Trace = 0.609; partial η^2^ = 0.30. When the results for the intervention practices (dependent variables) were considered separately, significant differences between professional groups in the four components were revealed. The magnitude of the differences was moderate to large for three components: comprehensive assessment; protocols, psychotherapy, and connectedness; and, clinical multidisciplinary approach. In the component family involvement, the magnitude of the differences found was small. These results with correspondent post-hoc tests are reported in Table 3.

#### 3.3.2. Specific Training on Suicide Intervention

There was a statistically significant difference between health professionals who had and who had not specific training in suicide on the combined dependent variables of intervention practices with suicidal people: F (4, 231) = 6.202, *p* = 0.000, Wilk’s Lambda = 0.903; partial η^2^ = 0.10. When the results for the dependent variables were considered separately, the components that reach statistically significant differences were comprehensive assessment and family involvement, as shown in Table 4. Health professionals with specific training on suicide are more likely to perform a comprehensive assessment and to involve patient’s family than the health professionals without training. Hypothesis 1 is partially confirmed. 

Regarding the relationship between professional group and specific training, significant differences were found between professional groups (χ^2^ = 14.65, df = 2, *p* = 0.001). General practitioners were the professional group with less specific training (5%), and psychiatrists were the professionals who reported having more suicide training (33%). The proportion of psychologists with training was 18%.

Two-way between-groups analyses of variance were conducted to verify if the professional group moderates the relationship between specific training and the practices components, indicating that there is not an interaction between the two independent variables—professional group and specific training—in their effect on the dependent variable—practices components. The interaction effect did not reach statistical significance: IF (risk assessment) (2, 230) = 0.122, *p* = 0.886; IF (protocols/connectedness) (2, 230) = 0.819, *p* = 0.442; IF (multidisciplinary clinical approach) (2, 230) = 0.329, *p* = 0.720; IF (family involvement) (2, 230) = 0.112, *p* = 0.894. The statistically significant main effects for professional group and for specific training were confirmed.

#### 3.3.3. Number of Patient Suicide Attempts

There was a statistically significant difference on the combined dependent variables of intervention practices with suicidal people, according to the health professionals specific experience with suicidal patient: F (8, 362) = 4.148, *p* = 0.000, Pillai’ s Trace = 0.168; partial η^2^ = 0.08. When the results for the dependent variables were considered separately, the differences in the Assessment component reach statistical significance: F (2, 183) = 7.649, *p* = 0.001, partial eta squared = 0.08. The remaining intervention components (protocols, psychotherapy, and connectedness; clinical multidisciplinary approach and family) were not related to the clinical experience with suicide attempters. 

An inspection of the mean scores and corresponding post-hoc tests of the assessment component indicated that health professionals with many patient suicide attempts (≥9) scored significantly higher in the comprehensive assessment component (M = 4.38, SD = 0.53) than health professionals with few (≤3) (M = 4.06, SD = 0.55; *p* = 0.002) and moderate (4–8) (M = 4.01, SD = 0.61; *p* = 0.003) patient suicide attempts. The actual difference in mean scores was moderate (η^2^ = 0.08). (Between health professionals who had few suicide attempters and who had moderate there were no differences). Hypothesis 2 was only confirmed for comprehensive assessment.

## 4. Discussion

This study identifies the practices that are more likely to be adopted by psychologists, psychiatrists, and general practitioners towards a patient who seeks help after a recent suicide attempt, allowing to know if health professionals use or not evidence-based clinical practices. In general, therapeutic approaches need to be assessed with research designs of higher quality and robustness. Even though some categories of clinical interventions for suicidal behaviors have evidence for reducing actual suicide death and/or the repetition of suicidal behaviors [41,42]. Restricting access to suicide means [28,41], fostering connectedness [26,27], and the treatment of depression (psychological and pharmacological) and other mental health conditions are among practices or approaches with strong evidence of efficacy [29,30,41]. Family-based interventions showed promising effects on suicidal ideation and/or suicide attempts, mainly regarding youth suicidality as compared with usual care [43,44,45]. There is also some evidence that follow-up actions directed at people who attempt suicide are effective in reducing suicide risk, thus health professionals should foster an appropriate chain of care [41,46].

### 4.1. Comprehensive Risk Assessment

The suicide risk assessment is among the most difficult clinical abilities that health professionals have to learn [9,47]. A competent clinical assessment of suicide risk must include a set of priority questions, namely about the desire of death, the existence of a suicide plan, and the availability of lethal means, which should be explicitly discussed with the patient [48,49]. In this study, the majority of health professionals (70% to 84%) revealed a high probability to explore these priority questions with suicidal patients. They appear to achieve a better performance than those from the study of Roush et al. [14], in which only 34% reported to approach lethal means, contrasting with the 70% of the present study. 

Also, the assessment of depression and other fundamental risk factors (e.g., previous suicide attempts, hopelessness, alcohol and drugs use, circumstances, and triggers) was rated as likely or very likely to be adopted by a high proportion of clinicians. These results differ from some published studies, which have been observing failures of health professionals to recognize suicide risk and to talk about the subject with patients [18,22,23,24], but they are consistent with those of Jobes et al. [8] that showed that health professionals’ practices tend to be in accordance with key procedures of assessment good practices. This adds new data about health professionals’ competence to assess patients who may be at suicidal behavior risk. However, given the crucial role of these questions to determine the imminent and short-term risk [50], the proportion of health professionals in the present study who do not consider these issues as a priority is not negligible (in mean about 25%). This shows the paramount need of increasing training about suicide risk assessment, accessible to a larger number of clinicians, and especially to general practitioners, once they were the less likely to perform a comprehensive risk assessment. This result is in line with a previous study [11], which found that general practitioners reported more difficulties than psychologists and psychiatrists in the assessment and management of suicidal patients. In the current public health care system in Portugal, as well as in other countries, GPs working in primary health care act increasingly as gatekeepers for mental health. The accessibility, the non-stigmatization, and the broad scope of primary health care promote that GPs who work at public health centres are the health professional first sought by most people when facing concerns about general or mental health, life or behavioural problems.

Moreover, results concerning the exploration of the meanings, of the expectations regarding suicidal behavior and of the feelings about surviving were also encouraging in that a high proportion of participants appear to recognize the patient’s personal narrative about his own suicidality as an important element for a collaborative assessment and the management of suicidality. This approach was advocated by various suicidologists, including AESCHI working group [49,51,52].

### 4.2. Protocols, Psychotherapy and Connectedness 

This study confirms that it is not likely that health professionals use formal instruments, which is in line with the few previous available data [8]. Formal instruments may be unknown to health professionals, due to the low level of training on the area and to the technical difficulties that are reported by professionals [11]. Another possible explanation is that health professionals perceive instruments and protocols as having little usefulness for suicidality assessment, as pointed out by Jobes et al. [8]. Another relevant aspect is related to the use of no-suicide contracts. Among the health professionals of this study, 37% consider the use of written prevention suicide contracts. In previous studies equal [20], inferior [14], and superior [19] percentages were found. Thus, as highlighted in previous studies [20], training on suicide prevention should approach the topic of NSC in order to increase health professionals’ awareness about the dangers of this tool and to enhance the knowledge about the use of safety plans, as an alternative. The formulation of a safety plan is advisable and recommended, establishing security steps in the case of increased levels of risk. This should be done within a collaborative work between patient and health professional [53,54].

This component also includes referral to psychotherapy. The majority of health professionals of the present study (74%) indicated psychotherapy as an intervention with a high probability of being recommended in suicidal cases. Existing evidence supports the efficacy of psychotherapy, namely cognitive behavioural therapy (CBT), in preventing suicidal behavior [30,55,56]. Even though the study conducted by Crawford et al., [57] found no evidence of the effect of psychosocial interventions on rates of suicide, psychotherapy, namely CBT, is recognized as an evidence-based method in the suicidality intervention [30,46].

Fostering connectedness, namely follow-up contacts, using technology-based methods appears to reduce suicidal behavior [26,27]. Health professionals revealed poor adherence to follow-up contacts using phone and new technologies (only 33% and 7% suggested mobile phone and internet), preferring the in-person contact and appear to conceive these contacts as integrated in the chain of care. Given the widespread usage and potentialities of technologies for health prevention [58], including suicidal behaviors prevention [59], it will be useful to approach the theme of follow-up contact modalities in suicide prevention training. Increasing the sense of social connectedness may be promoted by health professionals through remote contact or psychosocial interventions. Both modalities found encouraging empirical results [26,27,60]. Moreover, the importance of connectedness for the treatment of suicidality was theoretically grounded. The interpersonal-psychological theory of suicidal behavior [49,61] holds that to enhance connectedness is one of the mechanisms of recovery in suicidal crisis. Social connectedness can be defined as a construct that measures how people interact and come together, including the quantity and quality of social connections. It is related to the need of being accepted as a member of groups—e.g., family, friends, colleagues, that is, related to the belonging sense. Joiner’s theory posits the interplay between three fundamental constructs in the genesis of suicidal behaviour: belonging sense, burdensomeness, and fearlessness of death or physical pain [49,61]. This integrative-psychological model proposes that suicidal ideation results from the interaction of two perceived psychological states: the belonging sense and the feeling of being a burden to others—burdensomeness. Each of these states can independently produce the desire for suicide, however the interaction of both increases this probability According to this model, a low belonging sense and a high burdensomeness are the mediators between emotional pain and hopelessness (considered more general categories) and the suicidal ideation. The third construct is learned fearlessness about physical pain, physical injury, and death. This acquired capability explains the path from severe suicidal ideation to suicidal behaviour [49,61]. 

### 4.3. Clinical Multidisciplinary Approach 

A chain of care provided by a multidisciplinary team, allowing for an integration of pharmacological and psychological therapy, is recommended for the treatment of co-occurring psychopathological conditions [46]. Depending on professional group, health professionals tend to promote an integrated treatment with high to moderate probability. General practitioners seem to foster multidisciplinary care more than any other group. This result is coherent with the current public health system existent in many countries. In fact, primary health care is a critical setting and general practitioners are crucial professionals for the recognition of suicide risk, as well as for the referral to specialized mental care. However, it would be useful if the referral would also occur in the opposite direction (from mental health professionals to primary health care), in order to optimize the therapy compliance and to ensure a close and regular follow-up. 

### 4.4. Family

The involvement of the family should be promoted by health professionals [46]. In the present study, family involvement was only considered with moderate probability. However, an encouraging result was found: trained health professionals are more likely to involve the family in the treatment process than untrained clinicians. Moreover, when comparing to the few data available [14], in the present study, a higher proportion of professionals tend to involve family. Health professionals must take into account the quality of nature of relationships within the family before fostering the role and involvement of family, in order to safeguard an appropriate support. Violence or abuse within the family require a special care.

As expected, the results of this study are in line with previous studies that found significant associations between lower levels of difficulties and specific suicide-related variables, namely, having training in suicide and a higher experience with suicidal patients [11], and also between higher specific skills and the two aforementioned variables [7,12]. Thus, the present study adds further evidence that preventive policy should promote the increase of specific training and that the experiential-learning can be an effective methodology, as earlier advocated [7,11,62]. Another important finding of this study is that the current training did not seem to influence the propensity for a multidisciplinary intervention, nor the use of protocols and new technologies to promote connectedness and regular follow-up. A possible explanation for this might be that the current training on suicide intervention does not approach these issues.

There are some methodological limitations to this study that should be taken into consideration when drawing implications from these results. First, the sampling process cannot guarantee representativeness. However, a systematic bias is unlikely, since additional methodological procedures were used. Second, although the psychometric properties are promising, a deeper analysis of the questionnaire is required. 

## 5. Conclusions

The main findings of the study were: (1) In general, it is probable that health professionals perform a comprehensive assessment of risk. A chain of care through a multidisciplinary approach was also scored as likely. The involvement of the family in the therapeutic process was considered with moderate probability. Formal instruments and protocols, including no-suicide contracts, are unlikely to be use. (2) Psychiatrists were the most likely to undertake a comprehensive assessment, while General practitioners were the least likely to engage in risk detection and they valued the multidisciplinary approach most. Psychologists were the most likely to use instruments and protocols, to refer to psychotherapy and to promote connectedness through mobile phone and internet communication. (3) As expected, specific suicide-related variables, namely, having training in suicide and a higher number of patient suicide attempts, were associated to a higher probability to perform a comprehensive assessment. Moreover, health professionals with specific training on suicide were more likely to involve the patient's family than professionals without specific training. This study is encouraging because, in general, health professionals showed that it will be probable for them to use evidence-based practices when facing a suicidal patient. However, a not negligible proportion of health professionals seems to fail, reporting a low probability to adopt recommended fundamental practices.

In sum, the implications of the results are: (1) Increasing the training of health professionals in suicide prevention is necessary and useful and GPs should be the priority target group. (2) With regard to the syllabus of training, it should include formal tools, namely safety plans, connectedness, and follow-up modalities, and the issue of multidisciplinary chain of care. (3) With regard to the learning methodology, clinical cases discussion and experiential learning are advised. (4) At the health policy level, measures fostering the multidisciplinary chain of care should be implemented in order to decrease barriers that can hinder team approach. (5) Further research should investigate the influence of suicidal patient profiles on the practices of health professionals. It would also be useful to know the relationship between the current practices of health professionals and the difficulties that they feel in the clinical practice with suicidal patients. Further research should also identify specific variables—training-related and professionals' characteristics—that may moderate the relation between specific training and the adoption of recommended practices. 

## Figures and Tables

**Table 1:** Participants’ Characteristics.

Characteristics	*N*	%
Participants (*N* = 239)		
Psychologists	126	52.7
Psychiatrists (general and child)	53	22.2
General Practitioner	60	25.1
Gender (*N* = 239)		
Female	166	69.5
Male	73	30.5
Specific training in suicide prevention (*N* = 239)		
Yes	43	18.2
No	193	81.8
Experience with suicidal behaviours in clinical practice (*N* = 234) *		
Patient suicide attempt or serious risk of suicidal behaviour	195	83.3
Patient suicide	64	26.9
Number of patient suicide attempts (*N* = 186)		
Few ≤3	83	44.6
Moderate 4–8	43	23.1
Many ≥9	60	32.3
Work places (*N* = 239)		
Hospitals	77	32.3
Public health centres	72	30.3
Schools or colleges/universities	57	23.9
Community centres	27	11.3
Others (e.g., centres for drugs addiction treatment)	28	11.8

* The total number of health professionals who had experience with suicidal patients was 196, of which 195 had patient suicide attempts, 1 had patient suicide (without patient suicide attempts) and 63 had both. Ten health professionals didn’t specify how many patients with suicide attempts they had.

**Table 2:** Principal components of health professionals’ practices towards suicide attempters.

Component Label	Items–Intervention	Responses Likely or Very Likely to Be Adopted–Valid %				Components			
		Total	Psychol	Psychia	GPs	F1	F2	F3	F4
1. Comprehensive Risk Assessment	I ask about the lethal means used in the attempt	70.3	68.3	86.8	60.0	0.736			
	I try to understand the motives that triggered the attempt	92.9	93.7	98.1	86.7	0.710			
	I ask if he/she wants to die	72.8	67.5	86.8	71.7	0.699			
	I ask what he expected when attempting suicide	70.6	69.0	86.5	60.o	0.693			
	I ask how he/she feels about having survived	62.3	38.9	64.2	21.7	0.685			
	I explore the existence of an elaborate suicide plan	83.6	83.3	98.1	71.7	0.677			
	I assess the circumstances in which the attempt was carried out	83.3	86.5	90.6	70.0	0.662			
	I approach the theme of death	69.5	73.0	83.0	50.0	0.649			
	I assess the risk factors	85.8	88.1	88.7	78.3	0.644			
	I try to understand the meanings of the suicide attempt	93.3	96.8	92.5	86.7	0.643			
	I ask what reasons he/she has for living and for dying	63.1	61.1	66.0	64.4	0.628			
	I ask about prior suicide attempts	84.1	84.1	94.3	75.0	0.575			
	I ask questions about problems he may be experiencing	91.6	91.3	94.2	90.0	0.548			
	I try to understand how the patient usually solves his/her problems	72.0	83.3	77.4	43.3	0.547	0.516		
	I ask about the two days prior to the suicide attempt	50.7	50.8	64.2	38.3	0.545	0.412		0.424
	I try to understand if there is a non-solved or current mourning process	70.6	73.0	67.9	67.8	0.545			
	I try to find out at what time the suicide attempt was carried out	38.1	34.1	58.5	28.3	0.533			0.517
	I assess the hopelessness	66.1	68.3	83.0	46.7	0.514			0.388
	I ask about the alcohol and drugs consuming habits	77.0	69.0	96.2	76.7	0.501			0.464
	I assess depression	92.9	88.9	98.0	96.7	0.489			
	I ask about the family suicidal background	74.0	65.9	98.1	70.0	0.483			0.453
2. Protocols, psychotherapy and connectedness	I use specific suicidal behavior assessment instruments	23.0	36.5	7.5	8.3		0.835		
	I use specific intervention protocols for suicidal behaviors	30.9	46.0	20.8	8.3		0.835		
	I use formal instruments to assess suicide risk	26.4	38.9	5.8	18.3		0.754		
	I refer to psychotherapy	73.9	85.7	50.0	70.0		0.560		
	I set written suicide prevention contracts	22.4	35.7	9.8	5.1		0.546		
	I carried out a personality evaluation	59.0	61.1	64.2	50.0		0.513		
	I give a mobile phone number	33.0	40.5	30.2	20.0		0.378		
	I suggest using the internet to communication	7.1	8.7	5.7	5.0		0.306		
3. Multidisciplinary clinical approach	I refer to psychiatric counselling/monitoring	83.7	75.4	86.5	98.3			0.665	
	I advise a continued care plan	90.4	90.5	86.8	93.3			0.602	
	I refer to a colleague who is better prepared in this area	50.6	49.2	15.1	85.0			0.538	
	I refer to psychological counselling	75.6	83.2	57.7	75.0			0.504	
	I prescribe medication/I refer to a professional who can prescribe medication	67.7	69.8	69.8	61.0			0.497	
	I refer to the general practitioner	19.6	14.3	5.7	44.6			0.401	
	I try that the patient be hospitalized	26.3	14.3	39.8	43.3			0.385	
4. Family	I engage the family in the process	59.8	86.5	90.6	70.0				0.774
	I conduct a family interview	40.1	38.9	64.2	21.7		0.379		0.737
	I provide counselling to the family	43.1	38.9	49.1	46.7				0.731
	% explained variance (44.10)					23.91	9.75	5.65	4.80
	Cronbach’s alpha					0.915	0.806	0.575	0.794
	Mean inter-item correlation							0.2	

Notes: F = factor; Psychol = Psychologists. Psychia = Psychiatrists. GPs = General practitioners.

**Table 3:** Differences in practice components according to professional group.

Variables	Professional Groups						Post-Hoc (*p*-Values)		
	Psychol M (SD)	Psychia M (SD)	GPs M (SD)	F (2, 236)	*p*	η^2^	Psychol vs. Psychia	Psychol vs. GPs	Psychia vs. GPs
Comprehensive risk assessment	4.10 (0.55)	4.43 (0.51)	3.87 (0.55)	15.08 ***	0.000	0.11	0.001	0.022	0.000
Protocols, psychotherapy and connectedness	3.14 (0.71)	2.49 (0.59)	2.42 (0.70)	30.30 ***	0.000	0.20	0.000	0.000	0.837
Multidisciplinary clinical approach	3.62 (0.54)	3.43 (0.49)	4.04 (0.56)	20.12 ***	0.000	0.15	0.100	0.000	0.000
Family	3.43 (0.92)	3.72 (0.76)	3.26 (0.82)	4.24 ***	0.016	0.04	0.090	0.419	0.012

Notes: Psychol = Psychologists. Psychia = Psychiatrists. GPs = General practitioners. *** Significant level at 0.05.

**Table 4:** Differences on practices according to training.

Variables	Specific Training				
	Yes M (SD)	No M (SD)	F (1, 234)	*p*	η^2^
Comprehensive risk assessment	4.44 (0.51)	4.05 (0.56)	17.72 ***	0.000	0.07
Protocols, psychotherapy and connectedness	2.95 (0.64)	2.80 (0.78)	1.50 n.s.	0.222	0.01
Multidisciplinary clinical approach	3.58 (0.53)	3.70 (0.59)	1.60 n.s.	0.208	0.01
Family	3.78 (0.71)	3.39 (0.89)	7.22 ***	0.008	0.03

Notes: *** Significant level at 0.05, n.s. = not significant.

## Appendix A

**Table A1:** Intervention Strategies towards Suicidal Behaviours Questionnaire (ISBQ).

**1st Part—Professional and Social-demographic Characterization**									
**1. Gender**		Female ◯	Male ◯						
**2. Age**		___________	years						
**3. Years of practice**		___________	years						
**4. Professional Group**		Psychologist ◯	Psychiatrist ◯				General Practitioner ◯		
**5. Work place**		___________							
**6. District of work**		___________							
**Training**									
**7. I have specific training in the area of suicidology**							Yes ◯	No ◯	
**If your answer to question 10 was YES choose what type of training**							Yes	No	
Epidemiology							◯	◯	
Forensic Sciences							◯	◯	
Detecting and management of suicide risk							◯	◯	
Crisis intervention							◯	◯	
SOS hotlines							◯	◯	
**2nd Part—Practices towards suicidal patients** To what extent it is likely or not to adopt the following practices/intervention strategies when facing a patient who seeks your practice after a suicide attempt. Mark the option that suits your clinical experience best on a scale ranging from 1 (not likely at all) to 5 (very likely). Even though clinical practice may vary depending on each case, try to answer according to your general and most likely practice. Don’t give too much thought to your statements. Spontaneous answers are the most valid ones.									
**Intervention Strategies**			**Not Likely at All1**	**Not Very Likely 2**	**Somewhat Likely 3**		**Likely 4**		**Very Likely 5**
1. I ask about prior suicide attempts			◯	◯	◯		◯		◯
2. I assess depression			◯	◯	◯		◯		◯
3. I set written no-suicide/suicide prevention contracts			◯	◯	◯		◯		◯
4. I ask questions about problems he/she may be experiencing			◯	◯	◯		◯		◯
5. I ask if he/she wants to die			◯	◯	◯		◯		◯
6. I ask what he/she expected when attempting suicide			◯	◯	◯		◯		◯
7. I use formal instruments to assess suicide risk			◯	◯	◯		◯		◯
8. I engage the family in the process			◯	◯	◯		◯		◯
9. I assess the circumstances in which the attempt was carried out			◯	◯	◯		◯		◯
10. I refer/advise to psychiatric counselling			◯	◯	◯		◯		◯
11. I ask about the lethal means used in the attempt			◯	◯	◯		◯		◯
12. I approach the theme of death			◯	◯	◯		◯		◯
13. I advise a continued care plan			◯	◯	◯		◯		◯
14. I try to understand the meanings of the suicide attempt			◯	◯	◯		◯		◯
15. I give a mobile phone number			◯	◯	◯		◯		◯
16. I refer/advise to psychological counselling			◯	◯	◯		◯		◯
17. I try to find out at what time the suicide attempt was carried out			◯	◯	◯		◯		◯
18. I assess the hopelessness			◯	◯	◯		◯		◯
19. I provide counselling to the family			◯	◯	◯		◯		◯
20. I try to understand the motives that trigger the attempt.			◯	◯	◯		◯		◯
21. I ask about the alcohol and drugs consuming habits.			◯	◯	◯		◯		◯
22. I explore the existence of an elaborate suicide plan.			◯	◯	◯		◯		◯
23. I use specific intervention protocols			◯	◯	◯		◯		◯
24. I assess the risk factors			◯	◯	◯		◯		◯
25. I carried out a personality evaluation.			◯	◯	◯		◯		◯
26. I ask about the family suicidal background			◯	◯	◯		◯		◯
27. I ask what reasons he/she has for living and for dying.			◯	◯	◯		◯		◯
28. I refer to a colleague who is better prepared in this area			◯	◯	◯		◯		◯
29. I suggest using the internet to communication			◯	◯	◯		◯		◯
30. I prescribe medication/I refer to someone who can prescribe medication			◯	◯	◯		◯		◯
31. I refer/advise to the general practitioner			◯	◯	◯		◯		◯
32. I try that the patient be hospitalised			◯	◯	◯		◯		◯
33. I use specific suicidal behaviour assessment instruments.			◯	◯	◯		◯		◯
34. I ask about the two days prior to the suicide attempt.			◯	◯	◯		◯		◯
35. I ask how he/she feels about having survived.			◯	◯	◯		◯		◯
36. I try to understand if there is a non-solved or current mourning process			◯	◯	◯		◯		◯
37. I conduct a family interview.			◯	◯	◯		◯		◯
38. I try to understand how the patient usually solves his/her problems.			◯	◯	◯		◯		◯
39. I refer to psychotherapy.			◯	◯	◯		◯		◯
**3rd Part** **—Contact with Suicidal Behaviours in clinical practice**									
**1. Did (or do) you have any patient who has made one or several suicide attempts?**									
Yes	◯	How many patients/clients?		How long ago was the last case?					Years
No	◯								
**2. Have you had a patient suicide?**									
Yes	◯	How many patients/clients?		How long ago was the last case?					Years
No	◯								
**3. Have you ever had a patient representing a serious risk of suicide or suicide attempt even though he/she hasn’t carried it out?**									
Yes	◯	How many patients/clients?		How long ago was the last case?					Years
No	◯								

**Thank you very much for your collaboration and contribution!**
